# Chronic pancreatitis with multiple pseudocysts and pancreatic panniculitis: A case report

**DOI:** 10.1097/MD.0000000000010911

**Published:** 2018-06-01

**Authors:** Yuqing Gu, Zhuyin Qian

**Affiliations:** Pancreas Center, The Second Affiliated Hospital of Nanjing Medical University, Nanjing, P.R. China.

**Keywords:** chronic pancreatitis, multiple pseudocysts, pancreatic panniculitis

## Abstract

**Rationale:**

Pancreatic pseudocyst can present single or multiple, inside or outside the pancreas. Pancreatic panniculitis is a rare skin lesion in pancreatic disease patients. The purpose of this study is to report a case of chronic pancreatitis coexisting with multiple pseudocysts and pancreatic panniculitis.

**Patient concerns:**

A 46–year–old man with chronic pancreatitis presented multiple small cystic lesions inside the head of the pancreas and two large cystic lesions adjacent to the tail of the pancreas. The patient also developed subcutaneous nodules involving upper and lower limbs, hands, and lower abdomen bilaterally.

**Diagnosis:**

The patient was diagnosed with pancreatic pseudocyst and pancreatic panniculitis resulted from chronic pancreatitis.

**Interventions:**

Bile duct stent and pancreatic duct stent placement was performed endoscopicly.

**Outcomes:**

Panniculitis faded three weeks later and the pancreatic pseudocysts disappeared six weeks later.

**Lessons:**

Clinicians should be aware of the manifestation of multiple pancreatic pseudocyst and pancreatic panniculitis, and endoscopic transpapillary drainage may be a effective way in this scenario.

## 1 Introduction

Pancreatic pseudocyst is a common complication of chronic pancreatitis. It can present single or multiple, inside or outside the pancreas which is sometimes difficult to differentiate from pancreatic cystic tumor.^[1]^ Pancreatic panniculitis is a rare skin lesion in pancreatic disease patients.^[2]^ Herein, we report a case of chronic pancreatitis coexisting with multiple pseudocysts and pancreatic panniculitis.

## 2 Case report

**Figure 1:**
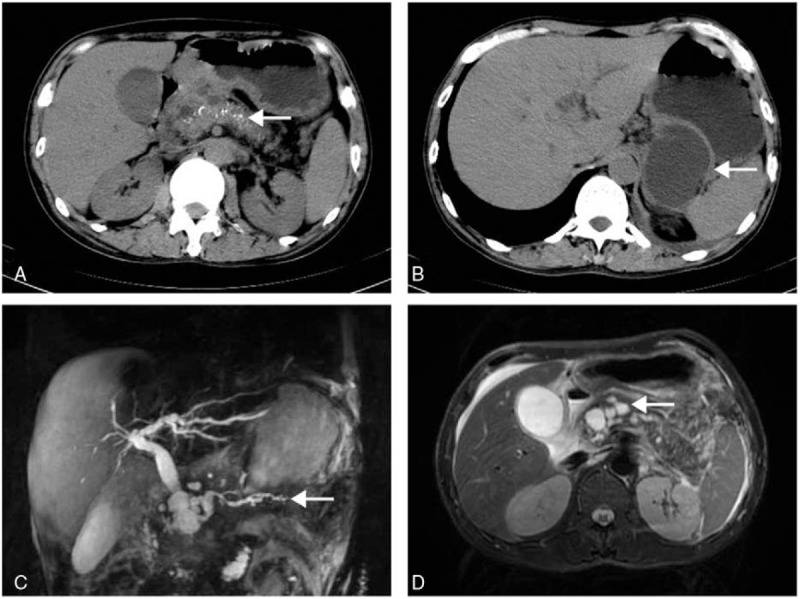
Abdominal CT and MR taken on admission. A, CT scan shows chronic pancreatitis and calcification of the whole pancreas. B, CT scan reveals a 6.5 × 5.7 cm cystic lesion next to the tail of the pancreas. C, MRCP shows irregular dilation of pancreatic duct and dilation of bile duct. D, MR scan reveals multiple small cystic lesions in the head of the pancreas. CT = computed tomography, MR = magnetic resonance, MRCP = magnetic resonance cholangiopancreatography.

In April 2017, a 46-year-old man was admitted to our hospital complaining of upper abdominal pain for 10 days. The patient reported a 3-year history of recurrent pancreatitis and a concurrent generalized body rash. The patient had a history of alcohol consumption and his father died of pancreatic cancer. On physical examination, there was direct tenderness but no rebound tenderness in the upper abdomen. No palpable mass, no rash was observed. Laboratory tests results: white blood cell count (WBC), 11.0 × 10^9^/L (normal, 4.0–10.0 × 10^9^/L); neutrophils proportion, 85.1% (normal, 50.0–75.0%); hemoglobin (HB), 108.0 g/L (normal, 120.0–160.0 g/L); serum total bilirubin (TB), 67.8 μmol/L (normal, 1.7–17.1 μmol/L); direct bilirubin (DB), 60.6 μmol/L (normal, 0–5.1 μmol/L); γ-glutamyltransferase (GGT), 454.1 U/L (normal, 10.0–71.0 μmol/L); the remainder of liver function tests were within normal limits; serum amylase (AMY), 883.4 IU/L (normal, 28.0–100.0 IU/L); tumor marker carbohydrate antigen 19–9 (CA 19–9), 30.8 U/mL (normal, 0–37.0 U/mL). In addition, abdominal computed tomography (CT) revealed pancreatic calcification, multiple small cystic lesions in the head of the pancreas, the biggest on measured 1.2 × 1.1 cm, and a 6.5 × 5.7 cm cystic lesion adherent to the tail of the pancreas. Magnetic resonance cholangiopancreatography (MRCP) showed cholecystitis, irregular dilation of pancreatic duct and dilation of bile duct, and demonstrated the same lesions of the pancreas as CT scan revealed (Fig. 1).

**Figure 2:**
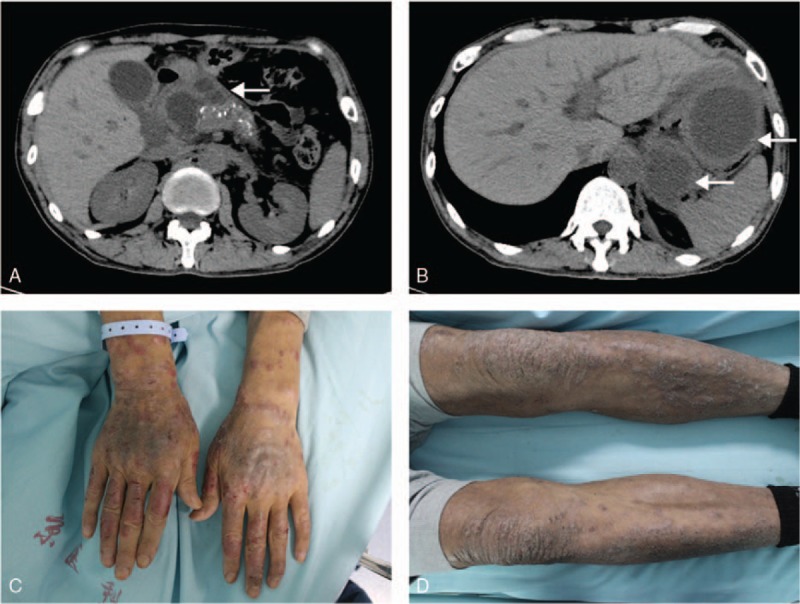
Results of abdominal CT and subcutaneous nodules 3 weeks after admission. A, CT scan shows the enlarged multiple cystic lesions in the head of the pancreas, the biggest 3.1 × 2.8 cm. B, CT scan reveals an 8.1 × 6.6 cm cystic lesion and a 5.2 × 4.1 cm cystic lesion next to the tail of the pancreas. C and D, erythematous, unclearly boundary, subcutaneous nodules of hands and lower limbs. CT = computed tomography.

The patient also suffered from pneumonia on admission and received levofloxacin 500 mg once daily according to the sputum culture and drug sensitivity test, and he was also under the treatment to pancreatitis including fasting, fluid resuscitation, and inhibition of secretion pancreas. Three weeks later, pneumonia was cured and the symptom of upper abdominal pain was relieved. Laboratory tests including blood routine examination, liver function tests, and serum amylase returned to normal. However, he developed rash erythematous, subcutaneous nodules with unclear boundary about 1.5 cm in diameter without heat involving upper and lower limbs, hands, and lower abdomen bilaterally. An abdominal CT reexamination showed that cystic lesions in the head of the pancreas became enlarged, the biggest measured 3.1 × 2.8 cm, and cystic lesion next to the tail of the pancreas grew bigger and separated into 2 chambers about 8.1 × 6.6 cm and 5.2 × 4.1 cm in size respectively (Fig. 2). The patient's blood glucose and serum glucagon was tested and the results were within normal ranges.

**Figure 3:**
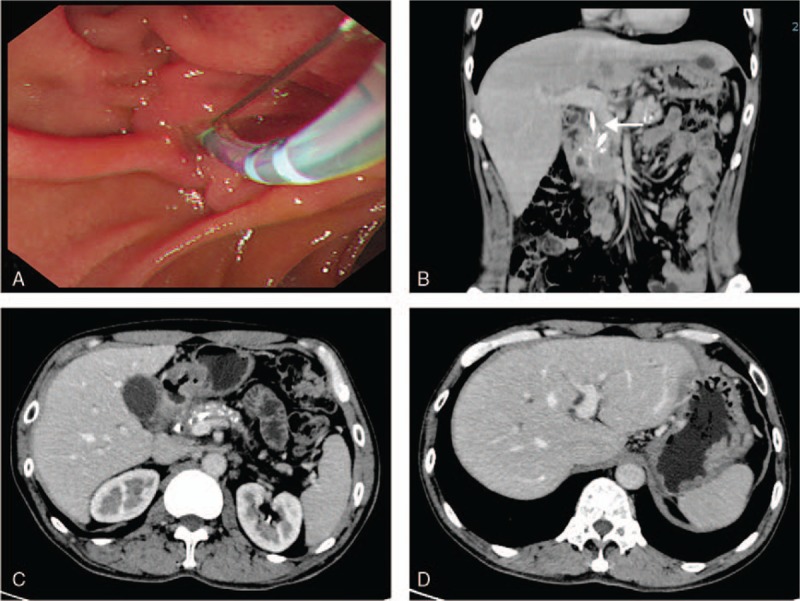
Intraoperative photo of endoscopic transpapillary drainage and abdominal CT taken after operation. A, ERCP shows opening of major duodenal papilla was normal. B, Coronal CT scan reveals the bile duct stent and the pancreatic duct stent. C, CT scan shows no cystic lesion in the head. D, CT scan reveals cystic lesion next to tail of the pancreas was disappeared 6 weeks after operation. CT = computed tomography, ERCP = endoscopic retrograde cholangiopancreatography.

Then, an endoscopic retrograde cholangiopancreatography (ERCP) was performed and showed that the opening of major duodenal papilla was normal and pancreatic juice was clear and transparent without mucus. Cholangiopancreatography depicted bile duct stenosis in intrapancreatic segment and proximal dilation, with duct stenosis in pancreatic head and distal dilation in the body and tail. A bile duct stent and a pancreatic duct stent was then placed. The patient received drug treatment after operation and recovered smoothly, abdominal pain relieved and the skin rash faded 3 weeks later. Laboratory tests including blood routine examination, liver function tests, and serum amylase were within normal ranges and abdominal CT reexamination showed that cystic lesion both in the head and tail of the pancreas reduced obviously 1 week later and disappeared 6 weeks later (Fig. 3).

The informed consent form of the patient is signed.

## 3 Discussion

Pancreatic pseudocyst is a common complication of chronic pancreatitis, reported the incidence rate ranges from 20% to 40%, and alcohol-related pancreatitis was found to be the most causative factor.^[3,4]^ Because of partial necrosis of pancreatic parenchyma and rupture of pancreatic duct, inflammatory exudate and pancreatic juice is wrapped up by fibrous connective tissues. Pancreatic pseudocyst is then formed by fluid collection lacking an epithelial lining, and it can present single or multiple, inside or outside the pancreas. The body and tail of pancreas resides retroperitoneumly, next to spleen and stomach, and inflammatory liquid can diffuse and accumulate at this space.^[5,6]^

The cysts inside the pancreas of chronic pancreatitis are often small, 2 to 3 cm in diameter, morphologically have no specificity in imaging findings and is quiet difficult to distinguish from pancreatic cystic tumors. The CT findings of pseudocyst are round or oval intrapancreatic or peripancreatic lesions, with water-like density, smooth cystic border, thin cystic wall with no enhancement in contrast scan, while cystic tumors are lesions with separated capsules, wall nodules protruding into the cavity, sometimes eggshell calcified cystic wall with slight enhancement.^[7,8]^ Our patient presented multiple pseudocysts inside and outside of the pancreas, which was difficult to distinguish from pancreatic cystic tumor according to CT and MRCP findings, the multiple small cystic lesions in the head of the pancreas mimics intraductal papillary mucinous neoplasm (IPMN) vividly.

Pancreatic panniculitis is a rare necrosis and inflammation of subcutaneous fat in patients with pancreatic disease. The probable mechanism is that lipase and amylase from damaged pancreas tissue enters to circulation and affects subcutaneous fat lobules.^[9]^ Pancreatic panniculitis typically presents as tender, edematous, and erythematous to red-brown subcutaneous nodules, most commonly involves lower legs, arms, and abdomen.^[10]^

It is less likely that pseudocyst of chronic pancreatitis can be absorbed spontaneously due to the complete maturation of the cyst wall, surgical or endoscopic intervention may be required.^[11]^ A surgical approach including internal and external drainage and excision of the cyst can be indicated in patients when endoscopic intervention failed or the patient has contraindications, or complications such as compression of the stomach or the duodenum, perforation, and hemorrhage. An endoscopic drainage can be achieved by transpapillary or transmural approach.^[12]^ Endoscopic transpapillary drainage is a good option for pseudocyst of chronic pancreatitis, because the pancreatic duct often communicates with the cyst, and a connection between the cyst and the duodenal lumen can be created by pancreatic duct stent. ERCP was performed on our patient and showed that pancreatic juice was clear and transparent without mucus, rules out the possibility of IPMN. Endoscopic transpapillary drainage was carried out and the cystic lesions both inside and outside of the pancreas were absorbed completely.

In conclusion, we report a male chronic pancreatitis patient presented with multiple small cystic lesions inside the head of the pancreas and 2 large cystic lesion adjacent to the tail of the pancreas, and subcutaneous nodules involving upper and lower limbs, hands, and lower abdomen bilaterally. Attentions should be payed to the manifestation of multiple pancreatic pseudocyst and pancreatic panniculitis of chronic pancreatitis patients. Endoscopic transpapillary drainage may be an effective way to some of these cases.

## Author contributions

**Project administration:** Zhuyin Qian.

**Writing – original draft:** Yuqing Gu.
